# Metformin acutely lowers blood glucose levels by inhibition of intestinal glucose transport

**DOI:** 10.1038/s41598-019-42531-0

**Published:** 2019-04-16

**Authors:** Olga Horakova, Petra Kroupova, Kristina Bardova, Jana Buresova, Petra Janovska, Jan Kopecky, Martin Rossmeisl

**Affiliations:** Department of Adipose Tissue Biology, Institute of Physiology of the Czech Academy of Sciences, Videnska 1083, 142 20 Prague 4, Czech Republic

## Abstract

Metformin is currently the most prescribed drug for treatment of type 2 diabetes mellitus in humans. It has been well established that long-term treatment with metformin improves glucose tolerance in mice by inhibiting hepatic gluconeogenesis. Interestingly, a single dose of orally administered metformin acutely lowers blood glucose levels, however, little is known about the mechanism involved in this effect. Glucose tolerance, as assessed by the glucose tolerance test, was improved in response to prior oral metformin administration when compared to vehicle-treated mice, irrespective of whether the animals were fed either the standard or high-fat diet. Blood glucose-lowering effects of acutely administered metformin were also observed in mice lacking functional AMP-activated protein kinase, and were independent of glucagon-like-peptide-1 or N-methyl-D-aspartate receptors signaling. [^18^F]-FDG/PET revealed a slower intestinal transit of labeled glucose after metformin as compared to vehicle administration. Finally, metformin in a dose-dependent but indirect manner decreased glucose transport from the intestinal lumen into the blood, which was observed *ex vivo* as well as *in vivo*. Our results support the view that the inhibition of transepithelial glucose transport in the intestine is responsible for lowering blood glucose levels during an early response to oral administration of metformin.

## Introduction

Metformin, the most potent of the biguanide analogs, was synthetized at the beginning of 20^th^ century and introduced to human medicine in 1958^1^. Due to its excellent abilities to manage blood glucose levels accompanied by a superior safety profile, metformin became the most widely prescribed drug for type 2 diabetes (**T2DM**)^2^. Moreover, in the guidelines for the treatment of T2DM published in 2012, the American Diabetes Association and the European Association for the Study of Diabetes recommended metformin for the initial treatment of T2DM^2^. The main advantage of metformin is its ability to reduce blood glucose concentrations in the long term, which is accompanied by improvements in insulin sensitivity of peripheral tissues without increasing the risk of hypoglycaemia^3^ or body weight gain^4^. Moreover, metformin has also shown benefits in reducing cancer risk and improving cancer prognosis^5^, which could be associated with the ability of metformin to negatively regulate aerobic glycolysis (i.e. the Warburg effect) in cancer cells and to suppress tumor growth *in vivo*^6^. However, despite 60 years of its extensive use the precise mechanism of metformin action is not sufficiently clarified.

It is widely accepted that the blood glucose-lowering effect of metformin is mediated mainly through the suppression of hepatic glucose production. Therefore, the liver has been considered as the primary site of metformin action, and several mechanisms have been proposed, which could mediate metformin’s action including the activation of AMP-activated protein kinase (**AMPK**) by the upstream liver kinase B1^7^, increased AMP/ATP ratio as a result of the inhibition of mitochondrial respiratory chain complex I^8^ and reduction of lactate and glycerol metabolism to glucose through a redox change by inhibiting mitochondrial glycerophosphate dehydrogenase^9^. Metformin could reduce hyperglycaemia also by improving peripheral insulin sensitivity and increasing glucose uptake in skeletal muscle^10,11^. Several studies suggest that the intestine also participates in blood glucose-lowering effect of metformin, mainly via changes in glucose uptake and anaerobic metabolism of enterocytes^12^, while increased production of an incretin hormone glucagon-like peptide-1 (**GLP-1**) could be involved as well^13^. Furthermore, there is convincing evidence that metformin changes intestinal microbiota in humans and the gut metabolome^14^, but the significance of this finding for whole-body glucose metabolism remains unclear. On the other hand, the activation of the glucose-lactate-glucose futile cycle during long-term treatment with metformin, which includes both the intestine and the liver, results in increased energy consumption^15^.

Long-term effects of metformin have been the main focus of the majority of studies so far, and only few studies focused on the mechanism associated with blood glucose lowering in response to acute administration of metformin^9,16,17^. With regard to the acute effects of metformin, it has been shown recently that intraduodenal infusion of this drug in obese and diabetic rats leads to a rapid (within the first 60 min) inhibition of hepatic glucose production during the clamp conditions, which is mediated via gut-brain axis, where the activation of duodenal AMPK represents the initial event^18^. It is also known that the activation of AMPK leads to an increase in GLP-1 levels, both acutely and in the chronic setting^16,18^. Moreover, the expression of GLP-1 receptor (**GLP1R**) is widely distributed throughout the brain including neurons producing N-methyl-D-aspartate^19^, and vagal afferent activation enhances N-methyl-D-aspartate receptor (**NMDAR**)-mediated neuronal transmission in the nucleus of the solitary tract to lower glucose production via the hepatic vagal branch^20^. Further in this context, a rapid blood glucose-lowering effect during an oral glucose tolerance test (**OGTT**) has been demonstrated in mice and rats after oral administration of metformin^16,21^, however no clear explanation for this effect was provided.

In the present study, we examined the mechanisms that mediate the acute blood glucose-lowering effect of orally administered metformin in obese C57BL/6J mice fed a high-fat diet (**HFD**) or in their lean counterparts. We aimed to identify the tissues that are involved in the effect of oral metformin administered to overnight fasted mice 30 min before the start of OGTT. Our results strongly implicate changes in glucose uptake and transport in the small intestine in the blood glucose-lowering effect of orally administered metformin and suggest that these effects are independent of functional AMPK, as well as of GLP1R and NMDAR signaling.

## Results

### Dose-dependent improvement of glucose tolerance in response to acute oral administration of metformin in high-fat diet-fed mice

**Figure 1:**
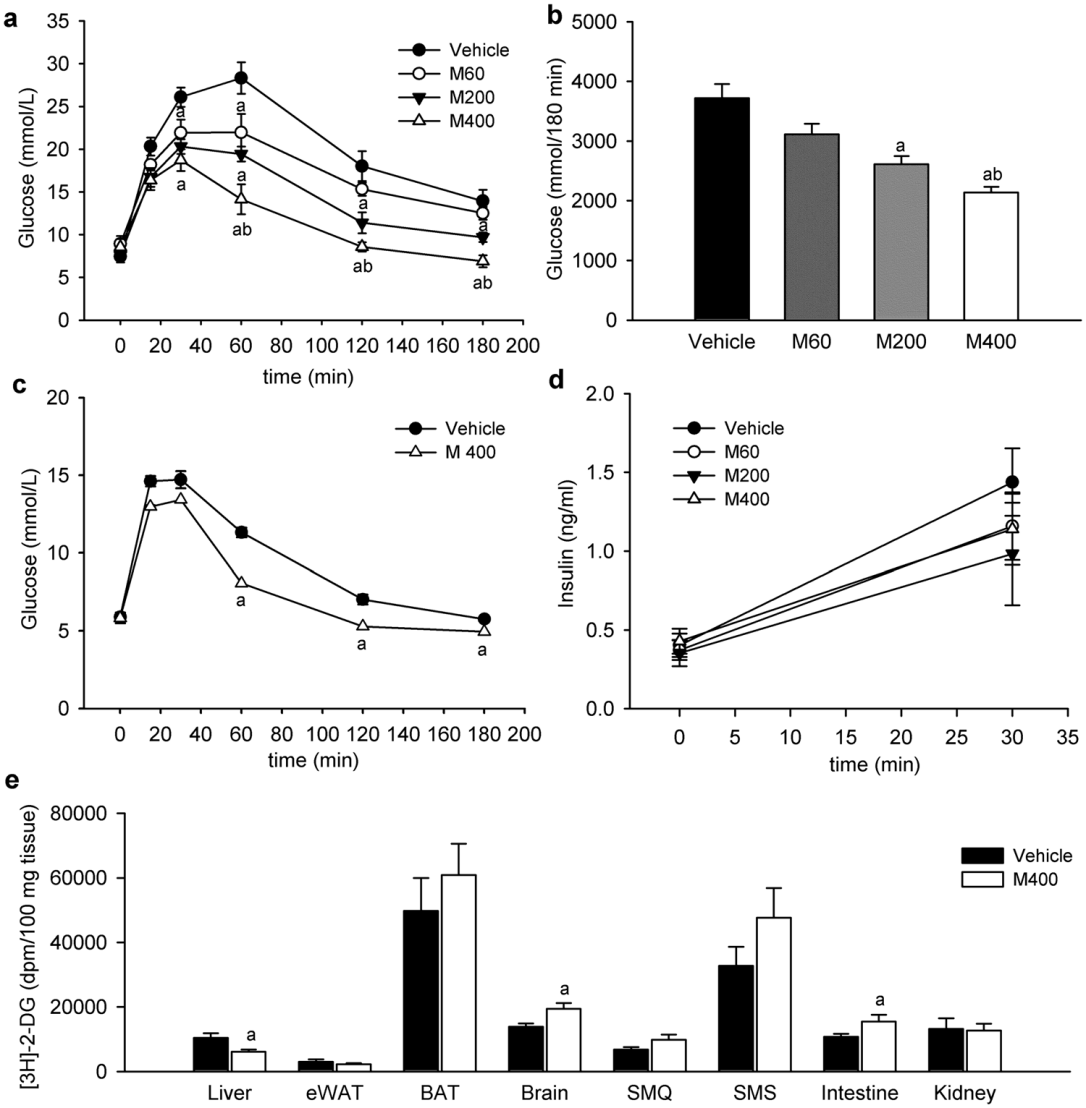
Metformin improves glucose tolerance independently of changes in plasma insulin levels. Overnight fasted mice fed HFD for 8 weeks were first given either vehicle or metformin at a dose of 400 mg/kg body weight (M400), 200 mg/kg (M200), or 60 mg/kg (M60) by oral gavage, and 30 min later D-glucose was administered either orally at a dose of 3 mg/g body weight or intraperitoneally at a dose of 1 mg/g body weight to start OGTT and IPGTT, respectively. **(a)** Glycemic curves during OGTT, and **(b)** the corresponding AUC values (a–b; ^a^P < 0.001 vs. vehicle; ^b^P < 0.015 vs. M60; One-way ANOVA. **(c)** Glycemic curves during IPGTT (^a^P < 0.005 vs. vehicle; t-test). **(d)** Plasma insulin concentrations during OGTT (One-way ANOVA). **(e)** Tissue uptake of [^3^H]-2-DG administered by *i.p*. injections to metformin (M400) or vehicle-treated HFD mice, and assessed 60 min after the injection of [^3^H]-2-DG. eWAT, epididymal white adipose tissue; BAT, brown adipose tissue; SMQ, skeletal muscle (*m. quadriceps*); SMS, skeletal muscle (*m. soleus*). ^a^P < 0.05 vs. vehicle; t-test. Data are means ± SEM (*n* = 6).

Male C57BL/6J mice were fed for 8 weeks a HFD starting at 3 months of age (see Table S1 in Supplementary appendix for basic characteristics). To evaluate the acute effect of metformin on glucose homeostasis, overnight fasted mice were gavaged with metformin at 400, 200 or 60 mg/kg of body weight (i.e. **M400**, **M200** and **M60**, respectively), and 30 min later they received glucose by gavage in the frame of a standard OGTT. Administration of a single dose of metformin resulted in the dose-dependent improvement of glucose tolerance as evidenced by decreased blood glucose concentrations during OGTT (Fig. 1a; P < 0.001). The level of glucose intolerance expressed as the total area under the glucose curve (**AUC)** was reduced in all metformin-treated groups as compared to vehicle-treated controls (Fig. 1b; P < 0.001). Moreover, the reduction in AUC caused by the highest dose of metformin (i.e. M400) was significantly greater than that caused by the lowest metformin dose (i.e. M60; Fig. 1b). To assess the impact of the route of glucose administration regarding the effect of metformin on blood glucose levels, overnight fasted mice were gavaged with M400 or vehicle and 30 min later they received glucose via* i.p.* injections in the frame of a standard intraperitoneal glucose tolerance test (**IPGTT)**. In contrast to OGTT, blood glucose levels did not significantly differ between the groups at 15 and 30 min after glucose administration (Fig. 1c). Of note, the change in plasma insulin levels that were determined at the baseline and 30 min after oral glucose administration was similar in all metformin-treated groups, thus suggesting that metformin-induced lowering of glycaemia during the OGTT cannot be explained by changes in plasma insulin levels (Figs 1d and S1).

**Figure 2:**
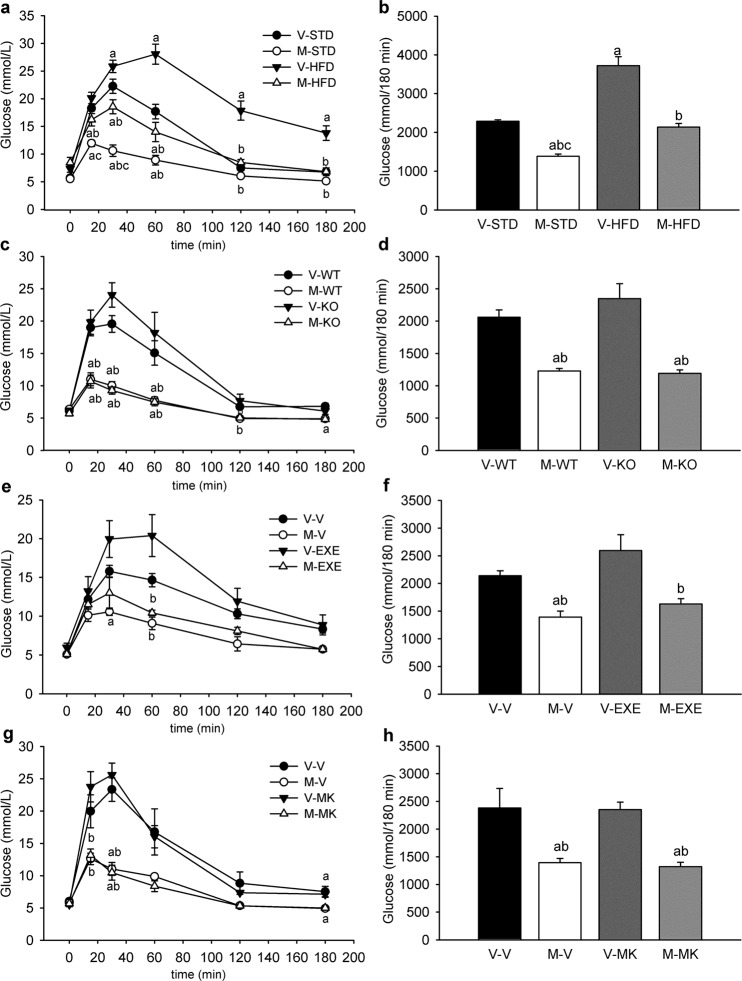
Metformin improves glucose tolerance independently of obesity, AMPK or GLP-1R and NMDAR signaling. Overnight fasted mice were given either vehicle (V) or metformin (M) at a dose of 400 mg/kg body weight by oral gavage, and 30 min later D-glucose was orally administered at a dose of 3 mg/g to start OGTT. **(a)** Glycemic curves during OGTT performed in STD- and HFD-fed mice treated with vehicle (V-STD and V-HFD mice) or metformin (M-STD and M-HFD mice), and **(b)** the corresponding AUC values. Data are means ± SEM (*n* = 6). ^a^P < 0.001 vs. V-STD; ^b^P < 0.001 vs. V-HFD; ^c^P < 0.001 vs. M-HFD by One-way ANOVA. **(c)** Glycemic curves during OGTT performed in AMPKα2-KO (KO) or wild-type (WT) mice treated with vehicle (V-KO and V-WT mice) or metformin (M-KO and M-WT mice), and **(d)** the corresponding AUC values. Data are means ± SEM (*n* = 6). ^a^P < 0.001 vs. V-WT; ^b^P < 0.001 vs. V-KO by One-way ANOVA. **(e)** Glycemic curves during OGTT performed in mice treated either with vehicle (V) or Exendin 9–39 (EXE), which were given via *i.p*. injections 10 min before either metformin (M-V and M-EXE mice) or vehicle (V-V and V-EXE mice) administration, and **(f)** the corresponding AUC values. Data are means ± SEM (*n* = 6). ^a^P < 0.005 vs. V-V; ^b^P < 0.005 vs. V-EXE by One-way ANOVA. **(g)** Glycemic curves during OGTT performed in mice treated either with vehicle (V) or MK-801 (MK), which was given via *i.p*. injections 10 min before either metformin (M-V and M-MK mice) or vehicle (V-V and V-MK mice) administration, and **(h)** the corresponding AUC values. ^a^P < 0.007 vs. V-V; ^b^P < 0.007 vs. V-MK by One way ANOVA. Data are means ± SEM (*n* = 4–5).

Dose-dependent improvement of glucose tolerance induced by metformin in the presence of similar plasma insulin levels suggest some alternative mechanism to lowering blood glucose levels in response to metformin. Thus, in order to understand the blood glucose-lowering effect of a single dose of metformin we performed 2-[1,2–3H (N)]-deoxy-D-glucose ([^**3**^**H]-2-DG**) uptake assay and examined the uptake of radioactively-labelled glucose from the blood into various tissues (Fig. 1e). M400 increased the [^3^H]-2-DG uptake in the brain and small intestine 60 min after [^3^H]-2-DG administration, while it unexpectedly decreased glucose uptake in the liver as compared to vehicle-treated animals (Fig. 1e; P < 0.05). Acute administration of metformin did not significantly affect [^3^H]-2-DG uptake in other major insulin-sensitive tissues such as adipose tissue and skeletal muscle when compared to vehicle-treated mice (Fig. 1e). Next, we examined whether the response to orally given metformin is also associated with glucose tolerance of STD fed animals. OGTT in a standard diet (**STD**) fed mice (see Table S1 in Supplementary appendix for basic characteristics) revealed that orally given metformin is able to lower blood glucose levels independently of the diet (Fig. 2a,b; P < 0.001).

### Glucose-lowering effect of acutely administered metformin is independent of functional AMPK and GLP1R or NMDAR signaling

In the following experiments, we tested whether any of the previously published mechanisms regarding the involvement of AMPK or GLP1R and NMDAR signaling could be involved also in the glucose-lowering effect of acutely administered oral metformin. To determine the role of AMPK, we performed OGTT in mice with a whole-body deletion of the α2 catalytic subunit of AMPK (**AMPKα2-KO**) mice and their wild-type littermates fed a low-fat chow, and also administered Compound C, a specific inhibitor of AMPK; the involvement of GLP1R and NMDAR signaling was then evaluated by examining the effects of their respective antagonists Exendin 9–39 and MK-801, respectively. In this context, metformin administered 30 min before the glucose gavage reduced glycaemia during OGTT in both wild-type and AMPKα2-KO mice (Fig. 2c,d; P < 0.001), as well as in mice that received Compound C (Supplementary appendix Fig. S2), Exendin 9–39 (Fig. 2e,f; P < 0.005) or MK-801 (Fig. 2g,h; P < 0.007). These data demonstrate that metformin-induced improvements in glucose tolerance are likely not mediated through the AMPK-GLP1R-NMDAR pathway activation.

### Reduced whole-body glucose oxidation during an early response to metformin

**Figure 3:**
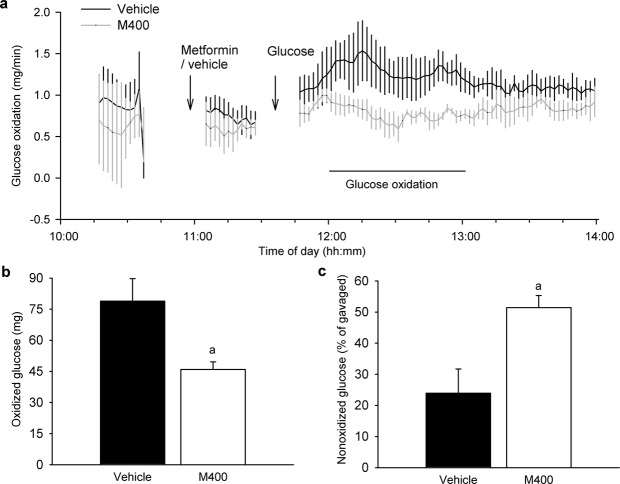
Whole-body glucose oxidation is reduced in response to oral administration of metformin. Overnight fasted mice fed HFD for 8 weeks were placed in the measuring chambers of the indirect calorimetry system INCA, and then given either vehicle or metformin at a dose of 400 mg/kg body weight (M400) by gavage. Thirty min later (i.e. at 11:30 a.m.) D-glucose was orally administered at a dose of 3 mg/g body weight. The use of substrates during the experiment was monitored as the change in the RQ values measured by INCA. **(a)** The curves representing changes in the whole-body glucose oxidation rate that was calculated from the data obtained by indirect calorimetry (see Methods for details); arrows indicate the administration times of metformin (vehicle) and D-glucose. **(b)** The amount of glucose oxidized during a time interval of 60 min (12:00 p.m.–13:00 p.m.) within the indirect calorimetry measurements. ^a^P = 0.021 vs. vehicle by t-test. **(c)** The amount of exogenous (i.e. administered) glucose that was not oxidized to CO_2_, calculated for a time interval of 60 min (12:00 p.m.–13:00 p.m.) during the indirect calorimetry measurements. ^a^P = 0.014 vs. Vehicle by t-test; Data are means ± SEM (*n* = 5).

Changes in tissue glucose uptake (Fig. 1e) cannot explain the above described decrease in blood glucose levels following an oral administration of metformin. Thus, we focused on the effect of metformin on whole-body energy metabolism. Indirect calorimetry measurements performed in overnight fasted animals treated orally with M400 or vehicle revealed no differences in the mean values of respiratory quotient (**RQ**) between the groups before glucose gavage (Supplementary appendix Fig. S3). In response to glucose administration, RQ values increased more in the vehicle-treated group, suggesting a higher glucose oxidation in mice without metformin. However, the RQ curves observed after glucose loading did not differ significantly between the metformin- and vehicle-treated groups (Supplementary appendix Fig. S3). The maximal achieved glucose oxidation rates were significantly higher in the vehicle (1.5 mg/min) as compared to metformin (1.0 mg/min) group (Fig. 3a), and corresponded to a significantly higher amount of oxidized glucose in the vehicle-treated animals measured during the 1-hour period following glucose administration (Fig. 3b; P = 0.021), which corresponded to pronounced differences in both RQ (Supplementary appendix Fig. S3). Conversely, in the metformin-treated mice, a greater portion of exogenous glucose was not oxidized as compared to vehicle-treated mice (Fig. 3c; P = 0.014). This could suggest that after metformin treatment there was an incomplete glucose oxidation to CO_2_ or glucose was not transported from gastrointestinal tract to plasma.

### Inhibition of transepithelial glucose transport from the intestinal lumen to the blood during an early response to metformin

**Figure 4:**
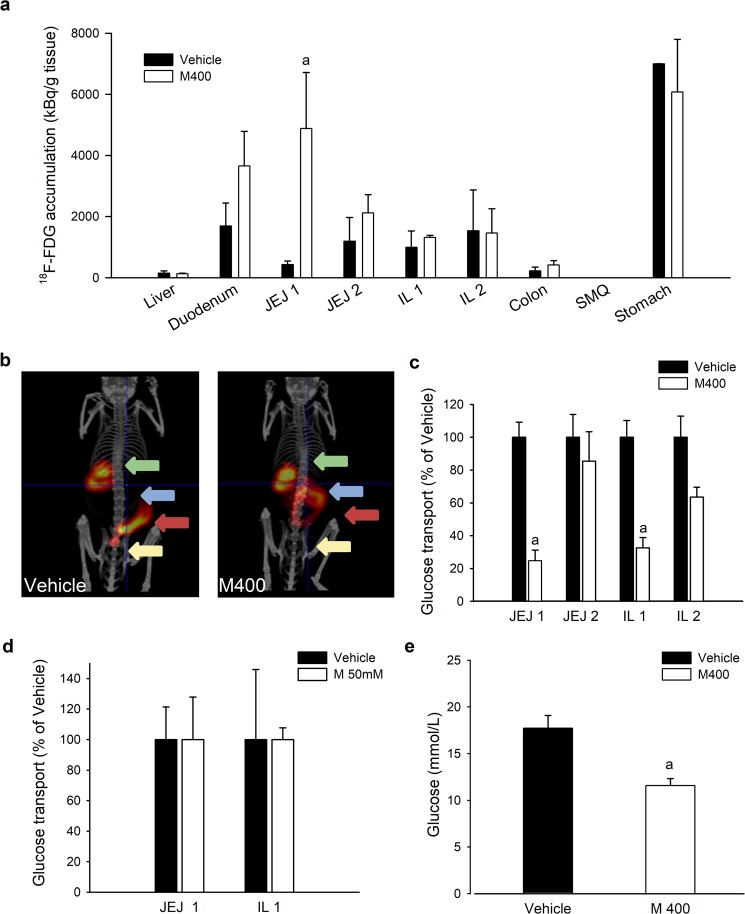
Metformin slows down the intestinal transit and stimulates glucose uptake from intestinal lumen into proximal intestinal segments while inhibiting glucose transport from intestinal lumen to circulation. Overnight fasted mice fed HFD for 8 weeks were first given vehicle or metformin at a dose of either 400 mg/kg (M400; **a–c,e**) or 60 mg/kg (M60; **e**) by oral gavage, followed by oral administration of [^18^F]-FDG (**a,b**) or incubation in 10 mM D-glucose solution (**c–e**) 30 min later. (**a**) The accumulation of [^18^F]-FDG in selected tissues measured during a time interval of 60 min following the administration of radioisotope. The intestinal content was carefully removed before the measurement. ^a^P < 0.005 vs. vehicle by t-test (**b**) Representative images of [^18^F]-FDG accumulation obtained by PET/CT scanning; the time frame is 20–30 min following the administration of [^18^F]-FDG; arrows: green, stomach; blue, proximal segments of small intestine; red, distal segments of small intestine; yellow, bladder. (**c**) Glucose transport across intestinal epithelia measured *ex vivo* using everted gut sacs prepared from mice previously treated with either metformin (M400) or vehicle. (**d**) Glucose transport across intestinal epithelia into serosal fluid measured *ex vivo* using everted gut sacs prepared from vehicle-treated mice, and in the presence of 50 mM metformin (M 50 mM) only in the medium. (**e**) Glucose levels measured *in vivo* in blood samples from portal vein 10 min after intraduodenal administration of 75 mg glucose in mice previously treated with either metformin (M400) or vehicle. JEJ1, proximal jejunum; JEJ2, distal jejunum; IL1, proximal ileum; IL2, distal ileum. ^a^P = 0.008 vs. vehicle; t-test. Data are means ± SEM (*n* = 6).

Based on our observation that anaerobic glycolytic activity in the small intestine is not altered by oral gavage with metformin (Supplementary appendix Fig. S4), we investigated the effect of metformin on the intestinal transport of orally administered glucose (Fig. 4). First, we performed PET imaging using radiopharmaceutical [^18^F]-FDG to visualize the accumulation of the tracer in the small intestine of mice pretreated with either M400 or vehicle (Fig. 4b); it showed that the intestinal transit of [^18^F]-FDG, assessed 30 min after its oral administration, was slower in metformin-treated mice than in vehicle-treated controls (Fig. 4b). The level of tissue [^18^F]-FDG uptake from the intestinal lumen, assessed 60 min after [^18^F]-FDG administration, was ~10-fold higher in the proximal small intestine of mice pretreated with metformin than in vehicle-treated controls (Fig. 4a; P < 0.005), while in the distal small intestine, colon and liver it was comparable between the two groups (Fig. 4a). In order to further analyze the transport capacity of the intestine for glucose, we performed *ex vivo* analysis of glucose transepithelial transport in the direction from the intestinal lumen to the blood using the technique of everted sacs prepared from different intestinal segments of mice pretreated either with metformin or vehicle (Fig. 4c). Glucose concentration in the serosal solution was almost ~3-fold lower when using everted sacs from proximal jejunum and proximal ileum of metformin-treated mice (Fig. 4c; P < 0.001; t-test), while in the sacs from distal jejunum and distal ileum glucose concentrations were comparable in both groups of mice (Fig. 4c). To examine whether metformin has a direct effect on glucose transport, everted sacs obtained from untreated mice were incubated for 60 min in the presence or absence of metformin (50 mmol/L). However, under these conditions, glucose concentrations in the serosal fluid were similar in both groups (Fig. 4d). To confirm the relationship between the reduced transepithelial glucose transport in the small intestine and blood glucose-lowering effect of acutely administered metformin, we tested whether the inhibition of intestinal glucose transport by metformin is also dose-dependent. *Ex vivo* analysis of glucose transepithelial transport in everted sacs prepared from mice that received either M60 or M400 revealed reduction of glucose transport in proximal jejunum by 28% and 70%, respectively, and in proximal ileum by 30% and 76%, respectively, when compared to vehicle-treated group (Fig. S5; P < 0.001). While the PET data may suggest not only slower intestinal transit but also delayed gastric emptying, possibly resulting in lower availability of glucose in the intestine of metformin treated animals, we bypassed the stomach through intraduodenal administration of glucose bolus 30 min after oral administration of metformin or vehicle. Glucose concentrations measured in portal vein blood 10 min later were significantly lower in metformin-treated mice (11.6 ± 0.8 mmol/L) as compared to vehicle-treated controls (17.7 ± 1.3 mmol/L; Fig. 4e; P = 0.008).

These data demonstrate that metformin stimulates glucose uptake from the intestinal lumen to the tissue primarily in the proximal jejunum, but at the same time reduces the transport of glucose to the blood across intestinal epithelia in the proximal jejunum as well as in proximal ileum in a dose-dependent manner. These metformin-driven changes in the intestinal glucose transport are apparent when metformin is administered by oral gavage *in vivo* but not when it is directly applied to intestinal tissue *ex vivo*.

## Discussion

We present evidence that the small intestine plays a key role in the early response to oral administration of metformin in mice. Metformin was more effective in lowering blood glucose levels when it was administrated orally as compared to its intravenous administration. In agreement with this observation, we demonstrate that within 60 min after oral administration of metformin transepithelial glucose transport in the proximal segment of both jejunum and ileum is inhibited, presumably via indirect manner. We further demonstrate that the acute effects of orally administered metformin in terms of blood glucose-lowering are independent of functional AMPK, as well as of GLP1R and NMDAR signaling.

Long-term effects of metformin with regard to regulation of glucose homeostasis are based on several distinct activities in various organs and tissues, which include increased glucose disposal, particularly in the skeletal muscle^22^, as well as reduced hepatic glucose production^9,23^. However, in our current study, peripheral uptake of intraperitoneally injected [^3^H]-2-DG, a non-metabolizable analogue of glucose, increased significantly only in the brain and small intestine during the early phase of acute response to oral metformin in fasted HFD mice. In this respect, an intense and diffuse accumulation of intravenously injected [^18^F]-FDG has been previously observed in the small intestine and colon in humans, which was interpreted as a physiological response to both short- and long-term treatment with metformin^24,25^. Furthermore, glucose uptake in enterocytes from the blood was increased ~2-fold in the small intestine and ~3-fold in the colon of metformin-treated humans and mice, which was associated with improved glycemic control^26^. In our study, we observed a ~10-fold increase in [^18^F]-FDG uptake from the intestinal lumen of the proximal part of the small intestine, which was assessed 60 min after oral administration of metformin and 30 min after oral administration of [^18^F]-FDG. This suggests that intestinal glucose uptake both from the intestinal lumen and from the circulation is an important determinant of the early-phase of metformin action. Increased glucose utilization in the intestine may contribute to metformin-induced lactic acidosis. Our findings that oral metformin administered at 400 mg/kg to fasted HFD mice did not induce an increase in lactate production is in agreement with the unchanged plasma levels of lactate after the intragastric administration of metformin at 300 mg/kg, which was reported previously in normoglycemic rats^27^.

Our current data suggest that reduced transport of intragastrically administered glucose across intestinal epithelia might be responsible for blood glucose lowering in response to oral metformin administration. This conclusion is based on the calculation of whole-body glucose oxidation, which was derived from the data obtained by indirect calorimetry, as well as on the fact that lactate production was not changed after oral metformin. An important role of the intestine is also supported by the fact that the strongest glucose-lowering effect of metformin is observed after its oral administration as compared to intraportal or intravenous infusions^28^. Following an oral administration of metformin in humans, ~70% of the dose is absorbed from the small intestine while the remainder passing into the colon before being excreted in feces^29^. Non-metabolized metformin is also excreted in urine, since no metabolites have been reported^29^.

We found that the acute response to oral administration of metformin followed by orally given glucose includes a profound inhibition of glucose transport from the intestinal lumen to circulation in the proximal segment of both jejunum and ileum. The difference between the uptake of [^18^F]-FDG into tissue *in vivo* and glucose transport in everted gut sacs of proximal ileum is presumably caused by different glucose availability. Thus, in postprandial state, glucose passing through the intestine reached low concentrations in the ileum, whereas all the gut segments that were analyzed as the everted sacs *ex vivo* were incubated in the solution of equally concentrated glucose. Glucose absorption from lumen to enterocytes is known to be mediated by Na(+)/glucose co-transporter (also known as SGLT1) that is located on the apical membrane of the cells, while the passive exit of glucose across the basolateral membrane occurs through the uniporter glucose transporter 2 (**GLUT2**)^30^. Mutations in SGLT1 cause a major defect in glucose absorption, but mutations in GLUT2 do not appear to disrupt glucose absorption^31^. Studies on Fanconi-Bickel patients with a mutation in GLUT2 suggest that there is another exit pathway for glucose that may involve exocytosis^32^. Little is known about how metformin regulates GLUT transporters, although some evidence shows that metformin promotes their translocation to the apical membrane^33^. It is also reported, that metformin stimulates expression of SGLT1 and GLUT2^34^. Recently, a polymorphism in the Slc2a2 gene, associated with altered GLUT2 expression in the small intestine, liver and other tissues, was identified as a genetic component of the glycemic response to metformin using genome-wide association studies^34^. We showed that metformin-induced inhibition of the intestinal glucose transport to circulation, together with the observed increase of intestinal glucose uptake into enterocytes, lead to accumulation of glucose in the intestine. This accumulation could be either a result of inhibited glucose transport to circulation or an impairment of glucose metabolism in enterocytes.

Lack of an inhibitory effect of metformin on the intestinal transepithelial glucose transport observed when metformin was applied directly to everted sacs obtained from different intestinal segments of previously untreated mice suggests that this process is regulated indirectly and is dependent on the oral route of metformin administration. Since metformin promotes apical localization of GLUT2 via activation of AMPK in rodent enterocytes^35^, and the inhibition of hepatic glucose production has been shown to be driven by the duodenal AMPK-GLP1R-NMDAR-dependent pathway^18^, we tested the role of AMPK in the acute response to orally given metformin. While the AMPK α1/β2/γ1 complex is more abundant in differentiated intestinal epithelial cells^36^, the AMPK α1 deletion in intestinal epithelium suppressed intestinal differentiation in mouse jejunum with a reduced mucosal height and villin content^37^. In contrast, mice lacking AMPKα2 are characterized by decreased protein levels of GLUT2 and GLUT5 in the intestine, and increased levels of SGLT1 in the jejunum^38^. In our current study, in response to acute metformin treatment, the AMPKα2-KO mice exhibited an equal suppression of blood glucose levels during OGTT as wild-type mice. Furthermore, since neither Compound C, Exendin 9–39 nor MK-801 affected the ability of metformin to lower blood glucose levels during OGTT, the involvement of the AMPK-GLP1R-NMDAR pathway in the inhibition of intestinal glucose transport during the acute response to oral metformin can be excluded. However, our results do not rule out other possible mechanisms involved in the blood glucose-lowering effect of oral metformin, such as the inhibition of hepatic glucose production (**HGP**) driven by the gut-brain-liver axis^18^. Nevertheless, inhibition of HGP appears to be less involved in acute lowering of blood glucose levels in response to metformin when compared to inhibition of intestinal transepithelial glucose transport in the intestine, since (i) we did not observe differences in blood glucose levels 30 min after administration of metformin (i.e. before the start of OGTT) as compared to vehicle-treated mice, (ii) we observed a stronger blood glucose lowering effect of metformin when glucose was administrated orally as compared to* i.p.* administration, and (iii) the expected effects of metformin on HGP during OGTT should be more profound in HFD mice with impaired insulin-driven HGP inhibition when compared to insulin sensitive STD mice, but this was not the case.

Our observation, that metformin slows down gastric and intestinal passage of FDG, implicates a delayed gastric emptying or decreased intestinal motility as a possible indirect mediator of metformin’s action. The rate of gastric emptying is now recognized to be a major determinant of the early as well as the overall postprandial glucose excursions in both healthy individuals and T2DM subjects^39^, while the acute oral metformin administration was shown to slow down gastric emptying in both the diabetic and control mice^40,41^. The fact that intraduodenal administration of glucose did not diminish the blood glucose-lowering effect of metformin does not support the role of gastric emptying as the major mechanism involved. In this regard, a study in healthy humans using intraluminal impedance monitoring indicated that pharmacological reduction of intraduodenal flow with an anticholinergic agent is associated with delayed absorption of luminal glucose^42^. Reversely, a previous study has documented that stimulation of intestinal motility is associated with a rise in nutrient absorption in rats^43^. In both mice and humans, intestinal smooth muscle cells contractility in response to acetylcholine is mediated by M2 and M3 muscarinic receptors^44^. Previous study implicated the M3 muscarinic pathway in the mediation of the effect of metformin^45^. On the other hand, acute changes in the blood glucose concentration are now recognised to have a major, reversible impact on the motor function of the gastrointestinal tract^46^. The upper gut function appears to be modified by inputs from the central nervous system. Therefore, our finding that metformin increased glucose uptake also in the brain may suggest the role of CNS in the acute effect of metformin, probably at the level of glucose sensing and regulation of intestinal motility. It has been shown that acute metformin administration activated neurons in the paraventricular nucleus (**PVN**), area postrema, and central amygdala^47^. GLUT2, the more suitable candidate for brain glucose-sensing, is expressed in the PVN^48^, and glucose-excited as well as glucose-inhibited neurons are also located there. Furthermore, both types of neurons are located in the dorsal vagal complex in the brain stem, which encompasses the dorsal motor nucleus of the vagus^49^.

In conclusion, we show that the inhibition of segment-specific glucose transport from the intestinal lumen to the blood represents a common mechanism that is employed in the acute response to oral administration of metformin, which is independent of any changes of metabolism induced by obesity and/or insulin resistance. Our findings contribute to understanding of the general mechanisms by which metformin exerts its effects on glucose homeostasis, and which might also contribute to its anti-diabetic effects upon chronic administration.

## Methods

### Animals and experimental setup

Three-months-old C57BL/6J strain susceptible to development of obesity (from the colony maintained at the Institute of Physiology) as well as transgenic male AMPKα2-KO mice (see ref.^50^) and their wild-type littermates on C57BL/6J background were kept in a controlled environment, i.e. at 22 °C, 50% humidity, and 12-h light-dark cycle, with food and water *ad libitum*. Mice were fed either STD (Ssniff R/M-H diet, Ssniff Spezialdieten GmbH, Soest, Germany), containing 9 wt% as lipids, or corn oil-based HFD containing 35 wt% as lipids^51^ for 8 weeks. On the day before each experiment, food was removed from the cages for ~15 hours (starting at 6:00 p.m.). On the day of experiment, mice were randomly assigned to treatments at a number of 5–6 mice per group. The experiments followed guidelines for the use and care of laboratory animals of the Institute of Physiology which are based on the Czech law regarding the protection of animals against cruelty (no. 246/1992). The experiments were approved by the Ethical Committee of the Czech Academy of Sciences under the protocol no. 81/2016.

### Glucose tolerance test and administration of drugs

Overnight fasted mice were treated either with saline (vehicle) or M400, M200 and M60, respectively(Glucophage; Merck, France) administered by oral gavage. Thirty min later animals were given D-glucose either orally at a dose 3 g/kg or intraperitoneally at a dose 1 g/kg and subjected to GTT. The degree of glucose intolerance was quantified as AUC. Insulin levels were measured in plasma obtained from blood samples collected before and 30 min after glucose administration, using the Sensitive rat insulin RIA kit (Merck Millipore, USA). Compound C (20 mg/kg body weight; Abcam), Exendin 9–39 (0.1 mg/kg body weight; Sigma Aldrich) and MK-801 (0.3 mg/kg body weight; Sigma Aldrich), i.e. the pharmaceutical antagonists of the AMPK, GLP1R and NMDAR, respectively, were administered via* i.p.* injections 30 min (Compound C) or 10 min (Exendin 9–39, MK-801) before metformin.

### Glucose uptake

Tissue uptake of glucose after metformin administration was evaluated using [3H]-2-DG solution (1 mCi/ml, Perkin Elmer, USA). Overnight fasted mice were first given either M400 or vehicle by oral gavage and 30 min later the mice received D-glucose (3 g/kg) by oral gavage and [^3^H]-2-DG (4 µCi/mouse) via *i.p.* injection. After additional 60 min (i.e. 90 min after metformin/vehicle administration), mice were killed by cervical dislocation under diethylether anaesthesia and the brain, liver, skeletal muscle (i.e. *m. soleus* and *m. quadriceps femoris*), white adipose tissue from the epididymal fat depot, interscapular brown fat, small intestine, and kidney were dissected. Tissues were dissolved in Solvable tissue solubilizer (Perkin-Elmer, USA), radioactivity was measured by liquid scintillation counting, and the final results were presented as disintegrations per min (**DPM**) per 100 mg of wet tissue.

### Measurement of lactate production

The lactate production was measured in jejunal explants using the Seahorse XF24 instrument. For a more detailed description see the Supplementary appendix.

### Indirect calorimetry

Overnight fasted mice were given either M400 or vehicle by oral gavage and placed into the measurement chambers of the indirect calorimetry system INCA (Somedic, Sweden). The chambers were thermostatically controlled at 22 °C, the readings corresponding to V_O2_ consumption and V_CO2_ production were recorded every 2 min under a constant airflow rate (1000 ml/min). After 60 min representing the baseline measurement, mice were given D-glucose solution (3 g/kg) by oral gavage and returned to the measurement chambers. To assess fuel partitioning, RQ was calculated (RQ = V_CO2_/V_O2_). The level of whole-body glucose oxidation was calculated using the equation (4.55 × V_O2_) − (3.21 × V_CO2_)^52^. The amount of oxidized glucose was calculated from a 60 min interval starting 30 min after glucose administration. The amount of non-oxidized glucose was calculated as the amount of glucose delivered by gavage minus the amount of glucose oxidized.

### [^18^F]-FDG-PET imaging

Overnight fasted mice were treated with a single dose of M400 or vehicle administered by oral gavage 30 min prior to [^18^F]-FDG administration. Approximately 10 MBq of [^18^F]-FDG dissolved in 0.3 ml of vehicle was administered by oral gavage. To verify the net injected dose, the residual dose of radiopharmaceuticals in the syringe was measured after the injection. Mice were maintained under the isoflurane anesthesia during the accumulation and scanning periods. PET and computed tomography (**CT**) scans were obtained on a dedicated μ-PET Albira system (Bruker, USA). A 10-min PET image acquisition began at 20 min following oral administration of [^18^F]-FDG. A CT-image was acquired for attenuation correction purposes. Images were reconstructed using the software PMOD (PMOD Technologies Ltd., Switzerland). All scans were visually analyzed. Liver, skeletal muscle, stomach and various segments of the small and large intestines (free of stool) were immediately excised and measured for radioactivity by a gamma counter.

### Everted gut sacs

Overnight fasted mice were given M400 or vehicle by oral gavage. After 30 min mice were killed by cervical dislocation under diethylether anaesthesia and two segments of jejunum and two segments of ileum were prepared as everted gut sacs^53^. Each segment was approximately 2 cm long. Sacs were filled with KRB buffer (i.e. representing the serosal fluid; 118.5 mmol/L NaCl, 4.75 mmol/L KCl, 2.7 mmol/L CaCl_2_, 1.19 mmol/L KH_2_PO_4_, 1.09 mmol/L MgSO_4_*7 H_2_O, 25 mmol/L NaHCO_3;_ pH = 7,2) and incubated in oxygenated KRB buffer containing D-glucose (10 mmol/L) at 37 °C for 1 hour. After the incubation, serosal fluid from the sacs was drained into a tube and glucose concentration in the serosal fluid was assessed using the glucose oxidase kit (Erba-Lachema, Czech Republic).

### *In vivo* assessment of intestinal glucose transport

Overnight fasted mice were treated either with saline (vehicle) or metformin (Glucophage; Merck, France) at 400 mg/kg of body weight administered by oral gavage. After 30 min animals were given 75 mg of D-glucose by injecting into the lumen of the duodenum of mice anesthetized with isoflurane. Ten min later, concentration of glucose was measured in blood sample collected from portal vein.

### Statistics

Data are presented as means ± SEM. Statistical analysis was performed with SigmaStat software. Data were analysed by two tailed t-test or One way ANOVA followed by Holm Sidak test. Specific P values are stated in the text of Results section and in the legends to figures P < 0.05 was considered significant.

## Supplementary information

Supplementary appendix

## Data Availability

The datasets generated during and analysed during the current study are available from the corresponding author on reasonable request.
