# Supplementary material for: Metformin acutely lowers blood glucose levels by inhibition of intestinal glucose transport
Source: Sci Rep. 2019 Apr 16;9:6156. doi: 10.1038/s41598-019-42531-0 (PMC6468119; doi:10.1038/s41598-019-42531-0)
Supplement: Supplementary file 1 — Supplementary appendix [file 41598_2019_42531_MOESM1_ESM.docx]

Supplementary appendix for the manuscript:

Metformin acutely lowers blood glucose levels by inhibition of intestinal glucose transport

Horakova O.^a*^, Kroupova P. ^a^, Bardova K., Buresova J., Janovska P., Kopecky J. and Rossmeisl M.

Department of Adipose Tissue Biology, Institute of Physiology of the Czech Academy of Sciences, Videnska 1083, 142 20 Prague 4, Czech Republic

^a^ Equal contribution

**Corresponding author:*

Olga Horakova, Department of Adipose Tissue Biology, Institute of Physiology of the Czech Academy of Sciences, Videnska 1083, 142 20 Prague 4, Czech Republic, E-mail: [olga.horakova@fgu.cas.cz](mailto:olga.horakova@fgu.cas.cz), Telephone: +420 296443706, FAX: +420 296442599

**Methods:**

**Measurement of lactate production by intestinal explants**
The lactate production was measured on explant of proximal jejunum of mice either treated placebo or metformin (400mg/kg) by oral gavage 30 minutes prior dissection. The Seahorse XF24 instrument (Agilent, USA) was used performing standard Glycolytic stress test protocol modified for tissue explants. Islet capture microplate was used for measurement of the jejunal explants. Approximately 1 mm^2^ of jejunal explant was placed in each well containing DMEM medium (Sigma Aldrich D5648), supplemented with 2 mmol/L L-glutamine and 0.2% BSA was placed in each well. Then the explants were challenged with 10 mmol/L glucose (final concentration in well) to stimulate the glycolysis, then 10 umol/L oligomycin to stimulate the maximal rate of glycolysis and to stop the reaction 2-deoxyglucose (**2-DG**) was injected to well in final concentration of 100 mmol/L. The extracellular acidification rate (**ECAR**) representing lactate production was calculated using the Wave software (Agilent, USA). Viability of the explants was monitored by simultaneous measurement of oxygen consumption in the wells.

**Table S1 Basic characteristics of experimental animals

 HFD STD**

**Body weight initial** (g) 29.1±0.43 30.72±0.58

**Body weight final** (g) 35.4±0.86 30.26±0.61

**Weight gain** (g) 6.28±0.75 -0.46±0.26

**Energy intake** (kJ/day/animal) 63.19±0.05 63.57±0.83

**Fasting blood glucose** (mmol/L) 8.10±0.40 5.80±0.20

Body weight gain was calculated as a difference in body weight determined at the start and at the penultimate week of dietary interventions within each mouse cohort, when experiment was performed. Energy intake was calculated using the 24-hr food consumption data collected once weekly from the start till the penultimate week of dietary interventions.

**Fig. S1 Metformin does not modulate the increase in insulin levels after orally given glucose in mice.**  Overnight fasted mice were pretreated with oral metformin at different doses (60 mg/kg, 200 mg/kg and 400 mg/kg body weight) or placebo. After 30 minutes glucose was administered at a dose 3g/kg body weight. The bar graph shows difference between plasma insulin levels assessed at baseline and 30 min after glucose administration. Data are means ± SEM (n = 6).

ab

**A**

bc

a

**Fig. S2 Metformin improves glucose tolerance independently of AMPK signaling.** Overnight fasted mice were given either vehicle (V) or metformin (M) at a dose of 400 mg/kg body weight by oral gavage, and 30 min later D-glucose was orally administered at a dose of 3 mg/g body weight to start OGTT. (A) AUC values derived from the glycemic curves obtained during OGTT in STD-fed mice treated either with vehicle (V) or Compound C (CC), which were given via i.p. injections 30 min before either metformin (V-M and CC-M mice) or vehicle (V-V and CC-V mice) administration, and (B) the corresponding AUC only in metformin-treated mice, which was expressed as a percentage of the respective control group without metformin treatment. ^a^P<0.001 vs. V-V; ^b^P<0.001 vs. V-M; ^c^P<0.001 vs. CC-V by One way ANOVA. Data are means ± SEM (n = 6).

**Fig. S3** Respiratory quotient calculated from indirect calorimetry measurement suggests lower whole body glucose oxidation after glucose gavage in response to acute oral metformin administration at 400 mg/ kg. Overnight fasted mice fed for 8 weeks HFD (35% fat) were treated with oral gavage of either metformin or vehicle 30 minutes prior glucose administration (3 mg/g by oral gavage). t-test; Data are means ± SEM (n = 5 per group).

**Fig. S4** Orally given metformin doesn’t affect lactate production in jejunum of HFD mice. Representative ECAR measurements, representing lactate production, obtained from the Glycolytic stress test performed in jejunal explants obtained from vehicle- and metformin-treated HFD mice using the Seahorse XF24 Extracellular Flux analyzer. t-test. Data are means ± SEM (n = 4 per group).

ab

ab

**Fig. S5 Metformin dose-dependently inhibits glucose transport** **across intestinal epithelia.** Dose-dependent effects of oral metformin on glucose transport across intestinal epithelia was investigated using everted gut sacs prepared from fasted mice pretreated either with vehicle or two different doses of metformin (M60 and M400) by oral gavage 30 minutes before preparation of the sacs. JEJ1, proximal jejunum; IL1, proximal ileum. ^a^P<0.001 vs. Vehicle; ^b^P<0.001 vs. M60 by One way ANOVA. Data are means ± SEM (*n* = 6).
